# Tapping Stem Cells to Target AMD: Challenges and Prospects

**DOI:** 10.3390/jcm4020282

**Published:** 2015-01-29

**Authors:** Caroline Brandl, Felix Grassmann, Julia Riolfi, Bernhard H. F. Weber

**Affiliations:** 1 Institute of Human Genetics, University of Regensburg, Franz-Josef-Strauss-Allee 11, 93053 Regensburg, Germany; E-Mails: Caroline.Brandl@klinik.uni-regensburg.de (C.B.); Felix.Grassmann@klinik.uni-regensburg.de (F.G.); Julia.Riolfi@stud.uni-regensburg.de (J.R.); 2 Department of Ophthalmology, University Hospital Regensburg, Franz-Josef-Strauss-Allee 11, 93042 Regensburg, Germany

**Keywords:** stem cells, induced pluripotent stem cells (iPSCs), retinal pigment epithelium (RPE), age-related macular degeneration (AMD), disease modelling, drug screening, cell-based transplantation therapy, RNA-sequencing

## Abstract

Human pluripotent stem cells (hPSCs) are increasingly gaining attention in biomedicine as valuable resources to establish patient-derived cell culture models of the cell type known to express the primary pathology. The idea of “a patient in a dish” aims at basic, but also clinical, applications with the promise to mimic individual genetic and metabolic complexities barely reflected in current invertebrate or vertebrate animal model systems. This may particularly be true for the inherited and complex diseases of the retina, as this tissue has anatomical and physiological aspects unique to the human eye. For example, the complex age-related macular degeneration (AMD), the leading cause of blindness in Western societies, can be attributed to a large number of genetic and individual factors with so far unclear modes of mutual interaction. Here, we review the current status and future prospects of utilizing hPSCs, specifically induced pluripotent stem cells (iPSCs), in basic and clinical AMD research, but also in assessing potential treatment options. We provide an outline of concepts for disease modelling and summarize ongoing and projected clinical trials for stem cell-based therapy in late-stage AMD.

## 1. Introduction

Age-related macular degeneration (AMD) is the leading cause of severe visual impairment and blindness in Western societies. With a steadily increasing life expectancy, the number of people with AMD is predicted to further increase worldwide to almost 200 million in 2020 and to over 280 million in the year 2040 [1]. Thus far, treatment options are limited and only exist for the neovascular (NV) form of late-stage AMD [2,3], a condition characterized by sub-retinal neovascularization with detachment of the sensory retina and/or the retinal pigment epithelium (RPE) and hemorrhages followed by sub-retinal scarring [4]. Another sight-threatening form of late-stage AMD, known in its final manifestation as geographic atrophy (GA), presents as atrophic lesions involving a gradual degeneration and disappearance of the RPE and photoreceptor cells within the central retina. The proportion of GA *versus* NV in late-stage AMD is approximately 20%–35% *versus* 75%–80%. This shifts to a higher frequency of GA in the population beyond 85 years of age [5,6] and further emphasizes the impact of GA on health in ageing populations. It also underscores the need for an effective treatment regimen for the near future.

AMD is a complex disease with still unknown pathophysiology. Multiple factors have been linked to pathogenesis and progression of the disease [7]. Among these are age and smoking, two risk factors consistently revealing a strong association with any form of AMD [8,9,10]. Nutrition, particularly dietary antioxidants, reduce AMD risk, as well as the progression of the disease [11,12,13]. Notably, AMD is strongly influenced by genetics. Estimates of heritability, a measure reflecting the proportion of observed variation in a particular trait attributable to genetic factors, vary from 45% to 71% [14,15,16]. Specifically, genetic variants in the complement pathway have been implicated as a major genetic contributor to disease pathology, implying a crucial role of the innate immune system in AMD pathogenesis [14,17].

AMD pathology relates to the functional syncytium consisting of the neurosensory retina, the RPE and the choriocapillaris, including the interjacent extracellular matrix [18], although the primary location of initial lesions is suspected to be on the level of the RPE [18,19]. The lack of adequate cellular and animal models in AMD has greatly limited our understanding of the molecular mechanisms and pathways involved in the development and progression of the disease [20]. Recent developments in human pluripotent stem cell (hPSC) research are most promising and could provide cellular models eventually mimicking “a patient in a dish”. Indeed, patient-derived cells or tissues are as close to the endogenous cellular situation as currently possible.

Notably, the application of hPSCs has been promoted in the field of ophthalmic research for a number of reasons. First, the eye offers easy access to surgical approaches and post-interventional follow ups. Furthermore, the cornea provides an excellent window for monitoring disease and treatment processes with highly sophisticated non-invasive anatomical and functional tools available [21,22,23]. Moreover, the eye is less prone to immune rejection of transplanted cells and tissues owing to its immune-privileged situation [24], although this privilege might become extinct when the blood/retina barrier is compromised due to disease, as is the case in NV AMD [25]. Finally, the inherent amplification of signals in the visual system permits noticeable rescue effects on vision given a relatively small number of rescued or transplanted cells [26,27].

This review discusses the current status and future prospects of utilizing hPSCs for understanding the pathomechanisms underlying AMD, but also for its use in assessing potential treatment regimens. We give a brief summary of the various types of stem cells available, with a special focus on induced pluripotent stem cells (iPSCs). The iPSCs hold particular promises with regard to disease modelling, drug screening and cell transplantation therapies of numerous degenerative human diseases [28]. We describe the generation of iPSCs and their advantages, as well as their limitations. We further elucidate the potential and pitfalls of hPSCs for disease modelling of AMD by outlining existing and possible concepts. Finally, we highlight some of the ongoing and planed stem cell-based clinical trials for AMD.

## 2. Stem Cells: Numerous Types, Infinite Potential

The value of stem cells is highlighted by two distinct properties, specifically the capacity for: (i) unlimited self-renewal as a result of asymmetric cell division, where at least one of the daughter cells holds traits of stem cells; and (ii) retaining an undifferentiated state and a high potency of cell differentiation. The latter feature marks the difference between diverse types of stem cells available in the human body. Stem cells can be classified by their differentiation potential: *i.e.*, totipotent stem cells can differentiate into both embryonic and extra-embryonic tissue; pluripotent stem cells have the ability to form all embryonic tissues (ectoderm, mesoderm and endoderm); and multipotent stem cells are able to differentiate into a limited number of somatic cell types, dictated by the degree of the earlier differentiation commitment [26,29].

Stem cells can also be categorized according to their origin. For example, human embryonic stem cells (hESCs) are derived from the undifferentiated inner cell mass of an embryo in the blastocyst stage 4–5 days post-fertilization and pre-implantation. The first stable hESC lines in cell culture were established by Thompson *et al.* in 1998 [30]. hESCs proved to be pluripotent with differentiation capacities for endoderm, ectoderm, mesoderm and even for germ cells that potentially generate whole organisms [30]. These cells promise to be powerful tools for therapeutic purposes, and there are high hopes for their use in replacing damaged tissue in patients suffering from degenerative disease [31]. However, clinical applications of allogeneic (donor) hESCs still need to overcome limitations and safety concerns, such as the restricted efficiency of certain hESC lines to adopt the desired cellular phenotypes, genetic and phenotypic instability, risk of graft rejection due to immune response or cancer formation after transplantation by residual undifferentiated hESCs [26,31]. Nevertheless, recent safety data from the first clinical trials are promising [32]. Moreover, isolating hESCs from the inner cell mass inevitably leads to the destruction of the blastocyst, which raises a number of ethical issues greatly limiting the broad utilization of hESCs [26,31]. Alternative approaches, specifically for the isolation of cells from earlier stages of embryonic development without the necessity to consume the embryo, have been addressed. Such approaches have proven successful, but less efficient [33].

Human stem cells can also originate from fetal tissue, such as fetal RPE cells, and are considered multipotent [26]. Umbilical cord tissue is another source of multipotent stem cells that have the potency to develop into a variety of somatic cell types [26]. Adult stem cells, also known as tissue-derived stem cell populations (TSCs), are found in most adult tissues and are able to maintain and regenerate a given tissue for a lifetime. Generally, human TSCs (hTSCs) are in a growth-arrested state with a slow cell cycle, but can re-enter the cell cycle on demand (e.g., after tissue injury) and give rise to differentiating and highly proliferative progenitor cells [26,34]. Importantly, hTSCs are not diffusely distributed in adult tissues, but require a stem cell niche, a microenvironment that provides external factors necessary for maintaining stem cell properties and functions [26,34]. HTSCs can be derived from adult somatic cell sources, suggesting that there might be fewer hurdles to overcome for their clinical application. Although endogenous hTSCs may carry fewer risks than allogeneic cell transplants, one has to take into account that endogenous hTSCs might often be defective due to primary disease; thus, they may not be suitable sources for treating primary disease [34]. A rich supply of adult hTSCs is bone tissue, which contains both hematopoietic and mesenchymal stem cells, housed in the marrow and the stroma, respectively [26,29,34]. For further information on these special types of stem cells, their clinical impact and recently elucidated relationships, the reader is referred to Frenette *et al.*, 2013 [35]. Adipose tissue represents an alternative, abundant and easily accessible source of adult hTSCs with the ability to differentiate along multiple lineage pathways [36].

hTSCs have also been characterized from the adult human eye [37,38]. Well known are the limbal epithelial stem cells (LESC’s), which regenerate corneal epithelium throughout life and, thus, have potential for clinical applications in corneal diseases [39,40]. With regard to retinal degenerative diseases, including AMD, retinal progenitor cells (RPCs) are of particular interest. RPCs have been found in the immature human retina, where they represent an immature cell population that is responsible for the generation of all retinal neuronal cell types during development, including retinal supporter cells, such as the Müller glia [41]. RPCs represent not a uniform type of cells, but rather, a group of progenitor cells at different stages of incomplete differentiation. They have also been identified in the adult human post-mortem retina by phenotype and neurosphere generation [42]. RPCs reveal stem cell-like properties, such as self-renewal abilities *in vitro*, but with a restricted capacity to differentiate into defined retinal neurons [42,43]. Unlike mature photoreceptor cells, RPCs have been shown to be reasonably efficient at integrating into the degenerative host retina [27,43]. Likely, their further use is limited due to the rather impracticable method of isolation, requiring scarcely available fetal or post-mortem tissue.

In 2006, a novel type of pluripotent stem cells, named induced pluripotent stem cells (iPSCs), heralded a major breakthrough in the stem cell field with the expectation to have a significant impact on basic science, technology and clinical medicine [29,44,45].

## 3. iPSC: The Stem Cell of the Future?

iPSCs were initially established from mouse and subsequently from humans [44,45,46]. Two seminal scientific contributions delineated the successful reprogramming of adult human somatic cells into pluripotent cells highly resembling hESCs [44,45]. This was achieved by overexpressing four transcription factors, including OCT3/4, Sox2, KLF4, c-Myc or OCT4, SOX2, NANOG and LIN28, respectively. Human iPSCs (hiPSCs) exhibit the essential characteristics of hESCs with regard to morphology, proliferation, surface antigens, gene expression, epigenetic status of pluripotent cell-specific genes and telomerase activity. Furthermore, hiPSCs can differentiate into advanced derivatives of all three primary germ layers *in vitro* and in teratomas. Consequently, these cells can differentiate in any somatic cell type of the human body and serve as an unlimited source for defined human cells [44,45].

Since then, hiPSCs have been appreciated as a valuable cellular source for disease modelling, drug screening and cell-based transplantation therapy in human degenerative diseases [29]. Still, critical issues need to be addressed, as the detailed mechanisms underlying the reprogramming process during hiPSC generation are not well understood at present [47].

Overexpression of stem cell factors in adult somatic cells was originally achieved by integrating techniques making use of retrovirus [44] or lentivirus [45] vector systems. However, integrating vectors have a rather limited clinical application due to potential risks of persistent reactivation of intrinsic pluripotency and of genome integration of transgenes. This includes altered differentiation potentials of the target cells and insertional mutations, both of which may lead to treatment-associated pathologies. Moreover, c-Myc is known as a proto-oncogene, making its clinical use highly unlikely due to potential tumor formation [48]. Consequently, techniques were developed to allow the generation of transgene-free or integration-free hiPSCs [49]. Those approaches include: (i) the use of non-integrating vectors, such as Sendai virus, episomal vectors or minicircle DNA [50,51,52]; (ii) the excision of vectors after integration via the CRE/lox-P system [53]; (iii) DNA-free delivery of factors directly as proteins or mRNA [54,55]; and (iv) chemical induction via small molecules [56]. These alternatives were shown to be successful, but appear more complex in application [48,49].

Of note, the reprogramming efficiency of adult cells to hiPSCs is low and was initially reported with a frequency as low as 10^−4^% [44]. Even to date, further improvements and the use of non-integrating approaches do not fundamentally overcome these limitations. Small molecules can enhance efficiency and reduce the number of transcription factors required, although increased reprogramming frequency and hiPSC safety appear to follow a negative correlation. Clearly, large-scale applications of iPSC technology await the validation of sophisticated protocols that sufficiently balance these two important elements [48,57].

Despite current shortcomings and despite the fact that *in vitro* cellular models still deviate from endogenous *in vivo* situations, iPSC technology is gaining momentum in the era of personalized medicine with the prospect to establish individual, patient-specific cell lines. A rich supply of adult donor cells can regularly be obtained from patients by non-invasive techniques. Importantly, in the case of autologous transplantation, immune rejection is considered less problematic. By now, various sources of somatic cells have been used to generate hiPSCs, among them skin, hair follicle, muscle, adipose tissue, bone marrow, peripheral blood lymphocytes and epithelial cells from urine [29,58,59]. This raised the issue whether epigenetic marks may persist from the adult cell source in the undifferentiated state of the hiPSC. Indeed, bi-sulfite sequencing revealed significant differences in methylation patterns between hESCs and hiPSCs and even among different hiPSC lines from the same source [29,60,61]. In addition, hiPSCs reveal clonal variation, seem to acquire genomic mutations in addition to epigenetic modifications and may have a greater propensity for genomic instability than hESCs with a higher rate of point mutations [62,63]. Importantly, these genomic aberrations and point mutations occur despite the exclusion of c-Myc as the reprogramming factor and the use of non-integrating methods for transgene delivery [62,63,64]. Yet, little is known about the causes of these mutations, the impact of differences in chromosomal epigenetics and about their biological consequences [60,62,65,66]. Another potentially important issue when studying hESCs and hiPSCs as models of human diseases are the possible confounding effects of X chromosome inactivation [67,68,69,70,71,72]. Since reprogramming affects the nuclear genome and leaves the mitochondria unaltered, the extent to which an aged or altered mitochondrial genome will influence the properties of hiPSCs and their derivatives also remains to be evaluated [73]. Genomic instability is recognized as one important hurdle in the expanding field of stem cell-based therapies, and growing awareness of the risk factors associated with human genome plasticity strongly advocates for the use of extensive genetic screenings as a measure of quality control to attest to the safety of stem cell-derived products [74].

## 4. Disease Modelling of AMD: Current Status

In AMD pathology, the cell types of interest involve the vascular endothelium, the photoreceptors and the RPE, all of which are not readily accessible from the patient, but can be generated via hiPSC technology: vascular endothelium from hiPSCs was demonstrated to exhibit the rich functional phenotypic plasticity of mature primary vascular endothelium [75]. Significant progress was made to identify the developmental stimuli that drive hiPSCs differentiation to various neurons, including retinal neurons. For example, hiPSCs were differentiated into multi-layer eyecup-like structures with the typical features of human retinal precursor cells, including photoreceptor precursors [76]. In another study, hiPSC-derived rod photoreceptors exhibited immunocytochemical characteristics and electrophysiological properties close to endogenous cells [77]. For further reading on specific aspects of hiPSC application to retinal disease, the reader is referred to two excellent reviews by Cramer and MacLaren, 2013 [78], and Wright *et al.*, 2014 [79]. Of note, recent work has focused on generating retinal ganglion cells from hESCs and hiPSCs [80].

**Figure 1:**
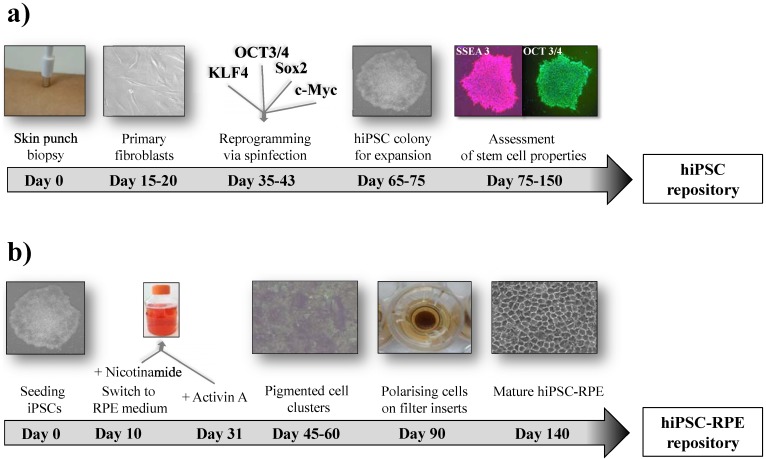
Representative timeline for the generation of skin biopsy-derived hiPSCs (**a**) and differentiated RPE cells (**b**). Major steps in the process are summarized. To obtain hiPSCs, integrating polycistronic lentiviral transduction via spinfection has been applied [90]. Due to the progress in the field of stem cell research, a number of integrating, but also non-integrating, protocols are available, and other sources than fibroblasts, such as blood lymphocytes, are widely used [29].

As the suspected cellular origin of primary AMD pathology, the RPE has attracted particular interest in the field of stem cell differentiation and *in vitro* modelling. RPE differentiation from hPSCs or hiPSCs is straightforward, as this cell type tends to differentiate spontaneously after removal of fibroblast growth factor (FGF) from the culture medium [81,82]. The ease of obtaining hiPSC-derived RPE cells is advantageous, as degenerative disorders involving the RPE are a common cause of visual impairment, highlighting the crucial role of this post-mitotic cell layer in retinal homoeostasis [83,84]. Several protocols for the direct differentiation of hPSCs into RPE cells have been established, and RPE cell cultures were reported to yield pure populations of functional cells that display many features of native RPE. Key parameters are addressed as the four “P’s” (polygonal, pigmented, polarized and phagocytic). Specifically, hexagonal cell morphology and pigmentation are pathognomonic for RPE cells. Moreover, functional features, such as transepithelial resistance or the polarized secretion capacities of known biological factors, like PEDF/VEGF, as well as photoreceptor outer segment phagocytosis, are essential characteristics of PRE cells. This is augmented by gene and protein expression of mature RPE markers [84,85,86,87,88,89,90,91]. In a note of caution, it was shown that highly differentiated, pigmented hiPSC-derived RPE monolayers can undergo only limited serial expansions before losing key cytological and functional attributes due to replicative senescence. This again underlines possible confounding effects of passaging cells as a general problem of cell culture disease models [88,90]. Addressing the limitation of serial expansion, Croze *et al.*, 2014, found that Rho-associated coiled-coil protein kinase (ROCK) inhibition allows for extended expansion of hESC-derived RPE cells. These cells remained functional for an enduring, but still finite, period of time in culture, possibly mitigating this problem [92]. An important aspect in terms of establishing cell repositories is the ability of hiPSC-derived RPE cells to regain viability and function after cryopreservation [90]. An exemplary timeline of a hiPSC protocol for generating RPE cells from biopsy material of adult skin is summarized in Figure 1. The reader is further referred to a sophisticated review by Bharti *et al.*, 2011. The authors emphasize the absolute necessity of providing an operational definition of a true RPE cell and offer a detailed list of testable criteria to monitor the molecular and functional authenticity of stem cell-derived RPE cells [91].

**Figure 2:**
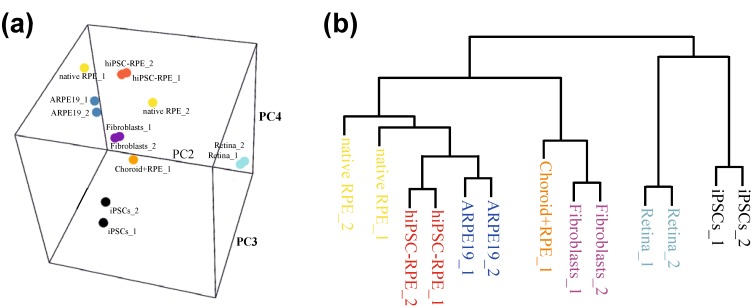
Deep RNA-sequencing and principal component analysis of different cell lines and tissues. (**a**,**b**) Deep RNA sequencing to analyze global gene expression profiles was performed for biological replicates of hiPSC-RPE cells, native RPE tissue, ARPE19 cells, RPE/choroid tissue, retinal tissue, hiPSCs and fibroblasts. Samples were clustered according to the main Components 2, 3 and 4. Results of the principal component (PC) analysis are given as (**a**) a 3D plot and (**b**) a phylogenetic tree. Comparison of global gene expression underlines the resemblance of hiPSC-RPE cells to native RPE tissue and indicates differences among native RPE tissue samples.

The validity of a hiPSC-derived RPE cell culture model greatly depends on its ability to mimic the behavior of native RPE cells in responding to normal and disease-associated stimuli. Consequently, hiPSC-derived RPE cells should reveal a set of expressed genes comparable to pure native RPE cells. A comparison of genome-wide expression profiles may provide a sensitive approach elucidating the differences and similarities in overall gene expression of two RPE lines. For example, by RNA sequencing, we compared a number of RPE lines, including hiPSC-derived RPE cells, an established RPE cell line, ARPE19 [93], native RPE/choroid tissue and retinal tissue. In addition, we analyzed the cell lineages used to generate the hiPSC-derived RPE, such as the dermal fibroblast cells and the hiPSCs generated thereof. The RNA reads obtained were aligned to reference sequences and quantified with tuxedo suite tools [94,95]. Principal component analysis grouped different cell types (and their replicates) according to their expression profile. This algorithm searches for genes with the highest rate of variation across all samples and groups the samples according to these genes. Our data reveal that independent cell lines and tissues from different donors have an overall high similarity in genome-wide gene expression (Figure 2a,b). Interestingly, native RPE tissue exhibits significant differences between its biological replicates (Figure 2a), which could be due to variation in the methods of tissue collection, post-mortem status or the variable degree of “contamination” of RPE with choroid or retinal tissue. This underlines that collecting native RPE tissue has numerous pitfalls and limitations for further (clinical) applications. Of interest, our data demonstrate a high similarity of hiPSC-derived RPE to native human RPE tissue, again underscoring the validity of hiPSC-derived cellular RPE models (Figure 2a,b).

**Figure 3:**
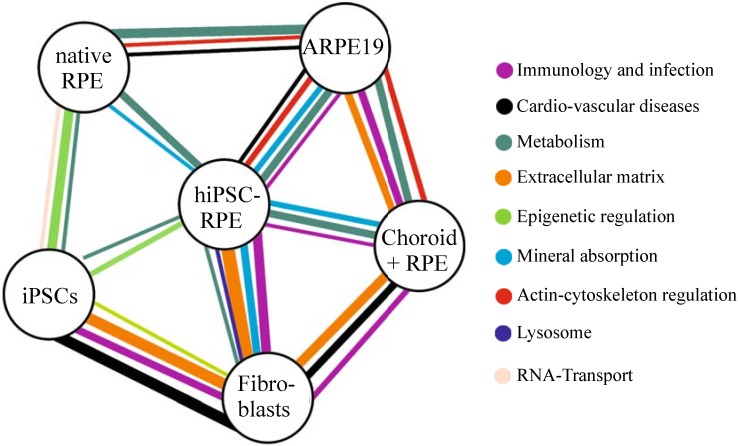
Kyoto Encyclopedia of Genes and Genomes (KEGG)-pathway analysis of different cell lines and tissues. Two hundred genes revealing the highest variances in RNA-Seq testing were selected and subjected to pathway enrichment analysis in the “G:Profiler” Software. Different colors code for different pathways. Broader lines indicate a lower *p*-value obtained from the pathway enrichment analysis.

A pathway enrichment analysis on differentially expressed genes points to those genes and pathways that mainly distinguish between the various cell types analyzed. To this end, two hundred genes with the highest variation between two different cell types/tissues were selected, analyzed with G:profiler software, and significantly enriched KEGG (Kyoto Encyclopedia of Genes and Genomes) pathways were recorded (corrected *p*-value <0.05). This demonstrates that genes with strong expression differences between iPSC-RPE cells and native RPE tissue are associated with only two pathways: mineral absorption (KEGG:04978) and general metabolism pathways (KEGG:01230, KEGG:00010, KEGG:01200, KEGG:00270) (Figure 3). In contrast, ARPE19 cells showed clear differences in several pathways to both native RPE tissue and iPSC-derived RPE cells. Together, these data show that hiPSC-derived RPE cells provide a cell culture model well in line with the native situation, not only morphologically and metabolically, but also in its global expression profile.

## 5. Disease Modelling of AMD: Future Possibilities

A recent study by Chang *et al.* [82] demonstrated that hiPSC-derived RPE cells from patients with GA due to AMD have a decreased antioxidative defense, making these cells susceptible to oxidative damage. Subsequently, curcumin, a potent ingredient of the spice plant, Curcuma longa, protected these cells from H_2_O_2_-induced cell death and also increased the cytoprotective effects against the induced oxidative stress. Notably, curcumin modulated the expression of several oxidative stress-regulating genes, which led the authors to conclude that this substance could be used as a drug effectively restoring RPE function [96]. This proof-of-concept study expertly illustrated the potential use of iPSC technology in future efforts to understand and treat AMD.

In combination with novel technologies to manipulate cell lines within a defined genetic background, such as CRISP/Cas9 editing of the genome [97], functional consequences of a single variant on the cellular phenotype can be delineated within a complex network of genetic risk and non-risk factors. In this context, it is of note that a complex late onset disorder, such as AMD, likely expresses a chronic low level pathology that is possibly influenced by differences in genetic background in addition to sequence variations in the disease-associated genes [98]. This makes it problematic when comparing hiPSC-derived RPE cells from a healthy control *versus* an affected individual, even when derived from siblings. Gene editing on a well-defined genetic background, e.g., via CRISPR/Cas9 editing [27,97], appears to be the method of choice and ideally allows assessing single AMD-associated sequence variants on a defined (high or low risk) isogenic background.

Functionally, the hiPSC-derived RPE cell lines from AMD patients can be analyzed in a great variety of cellular studies. Importantly, these studies can include responses of the RPE cells to challenges, including natural chronic stressors mimicking environmental risk factors associated with AMD, such as short-term and long-term photoreceptor outer segment (POS) feeding [99], activation of the complement cascade via human sera [17] or cigarette smoke simulated by cigarette extracts and nicotine [9,100,101]. Of note, Cano *et al.* expertly reviewed how cigarette smoke and oxidative stress to the RPE might contribute to AMD [102]. High throughput “omics” approaches to generate genome-wide transcriptome or metabolome profiles of AMD patient-derived hiPSC-RPE cells could help to define pathways in AMD pathogenesis. In turn, this could further our understanding of the consequences of a defined genetic variant and may elucidate local molecular mechanisms contributing to AMD pathology.

As mentioned previously, AMD pathology involves not only the RPE, but also immediately associated structures, such as the vascular endothelium and the photoreceptors. Therefore, it is tempting to consider future complex *in vitro* models that could include the RPE, human Bruch’s membrane (BM) and the subjacent choriocapillaris. This would allow expanding analysis from cells with simply having pathologic genetic alterations to investigate pathophysiological cellular interactions between the different cells types involved. Several models for co-culturing RPE with, e.g., endothelial cells have been described and may specifically be useful for studying NV AMD [103].

## 6. Cell-Based Therapy in AMD: Current and Projected Clinical Trials

Therapeutic applications of stem cells can be based on different strategies. For cell replacement therapy, stem cells are differentiated into the desired somatic cell type, which is then delivered to the diseased tissue in order to integrate and restore function [26]. An alternative approach uses the paracrine effect of transplanted stem cells, which secrete trophic factors that induce the resident tissue to self-restore and proliferate [26,104]. Additionally, there is some evidence that stem cells may fuse with individual existing cells in order to restore cellular function [26,104,105].

The focus in retinal stem cell-based therapy has been on replacement of photoreceptors and RPE. Substituting an RPE monolayer beneath the retina appears less complex than replacing retinal neurons, which need to integrate into the retinal network to ensure functionality [26]. Accumulating studies in animal models of retinal degeneration showed promising results [79], and in 2012, the first therapeutic stem cell application in a human clinical trial was reported by Schwartz *et al.* [106] with safe sub-retinal injections of hESC-derived RPE cells into patients with Stargardt disease and GA due to AMD. In a recent follow-up study including 18 participants, this group has again reported the safety of this therapeutic approach. In addition, the authors have also demonstrated improved vision in four out of nine AMD patients treated [32]. Interestingly, only few, if any, pigmented transplanted RPE single cells survived in the direct area of GA lesions. Instead, transplanted cells were detectable in areas adjacent to areas of GA, where they were deposited onto native RPE. Organ culture experiments also underline that aged and thickened submacular human Bruch’s membrane (BM) does not support long-term survival and differentiation of transplanted RPE [107,108]. Thus, if RPE transplants are meant to preserve and rescue high-acuity vision, developing strategies to improve transplanted RPE cell survival in areas of GA, typically adjacent to the fovea, will be crucial. Importantly, this implies that therapeutic application of stem cell technology for AMD may require not only development of the appropriate mature cell type, but also management of the extracellular milieu. Clinical cell-based transplantation trials have tried to overcome these hurdles by using RPE sheets instead of cell suspensions of disorganized RPE single cells [109,110]. RPE sheets can be grown on artificial scaffolds, which appear suited to replace the diseased BM [109]. On the downside, these techniques require a more complex surgical procedure; biodegradable materials might cause inflammation; while non-degradable membranes may separate the RPE from the underlying choroid that nourishes RPE and photoreceptors [110]. Table 1A,B provides an overview of current and projected clinical trials involving cell-based therapeutic approaches for late-stage AMD and summarises the main aspects of these studies. For more detailed information, references are provided.

HiPSC-derived RPE cells could emerge as valuable tools to explore potential treatment regimens. Proof-of-concept studies exist [96], but future concepts may want to emphasize molecular and functional differences between hiPSC-derived RPE cells from patients with low or high genetic risk for developing AMD. These differences could be targeted via large-scale drug screening experiments with a genotype-specific platform to define appropriate readouts. This could prove helpful in clinical trials to further promote personalized medicine in this blinding disorder.

**Table 1:** Phase I/II prospective safety (and efficacy) studies for stem cell-based therapy of late-stage AMD. (**A**) Integrating cell replacement strategies to engraft long-term and/or to functionally replace the degenerated endogenous RPE; (**B**) Non-integrating cell injections that mediate the effects by homing/modulating the inflammatory environment and/or releasing neuroprotective cytokines.

Study Centre	Year of Launch/Status	(Stem) Cell Type Used	Main Facts	Publications/Sources (NCT = ClinicalTrials.gov Identifier)
**A**				
Jules Stein Eye Institute at University of California Los Angeles (UCLA), USA; Advanced Cell Technology, Inc., Marlborough, Massachusetts, MA, USA	2011/preliminary report published in 2012	hESC-derived RPE suspension	▪ sub-macular injection via vitrectomy in one patient with Stargardt macular dystrophy and one patient with atrophic AMD ▪ hESC-derived RPE cells persisted for four months; no signs of hyperproliferation, tumorigenicity, ectopic tissue formation or apparent rejection ▪ vision improvement in patient with atrophic AMD from 21 Early Treatment Diabetic Retinopathy Study (ETDRS) letters to 28	Schwartz *et al.*, 2012 [106]; NCT01345006; NCT01344993
Multi center USA (Jules Stein Eye Institute at UCLA, Los Angeles, LA, USA; Bascom Palmer Eye Institute, Miami, FL, USA; Wills Eye Institute-Mid Atlantic Retina, Philadelphia, PA, USA; Mass Eye and Ear, Boston, USA); Advanced Cell Technology, Inc., Marlborough, Massachusetts, MA, USA	2011/report published in 2014	hESC-derived RPE suspension	▪ sub-macular injection via vitrectomy in patients with advanced Stargardt macular dystrophy and atrophic AMD ▪ enrolment of 18 patients in four study centers in the USA ▪ extension of the study above ▪ follow-up period for a median of 22 months ▪ no evidence of adverse proliferation, rejection or serious ocular or systemic safety issues ▪ increase in subretinal pigmentation consistent with transplanted RPE cells in 13 of 18 patients ▪ improvement in visual acuity of at least 15 ETDRS letters in eight of 18 patients ▪ increased vision-related quality-of-life measures	Schwartz *et al.*, 2014 [32]; NCT01345006; NCT01344993
University College London, Moorfields Eye Hospital, London, U.K.; Pfizer, Walton Oaks, U.K.	2007/stem cell transplantation trial approved in 2013, ongoing	hESC-derived RPE sheets	▪ transplantation of thin sheets of plastic polymer via vitrectomy in patients with neovascular AMD ▪ goal to overcome disadvantages of cell suspension ▪ currently preparing the transplantation cells/sheets *in vitro*	Carr *et al.*, 2013 [109]; NCT01691261
Riken Institute, Kobe, Japan	2013/ongoing	autologous hiPSC-derived RPE sheets	▪ sub-macular transplantation to neovascular AMD patients after surgical removal of choroidal neovascularisation (CNV) ▪ GMP-grade cell-processing facility ▪ pilot safety study, enrolment of six patients (estimated), follow-up for three years ▪ Nakano-Okuno *et al.*, 2014 [111], describe risk-benefit analysis	Kamao *et al.*, 2014 [110]
**B**				
Hollywood Eye Institute, Cooper City, Florida, FL, USA; Bioheart, Inc., Sunrise, Florida, FL, USA	2013/completion 2016 (estimated)	autologous adipose-derived stem cells (ASCs)	▪ intravitreal injection in atrophic AMD patients ▪ ASCs derived via liposuction; primary outcome measures: adverse events, visual acuity, visual field analysis	NCT02024269
University of California; Davis Eye Center, Sacramento, California, CA, USA	2012/completion 2014 (estimated)	autologous CD34+ bone marrow stem cells (BMSCs)	▪ Intravitreal injection in retinal degenerative conditions (atrophic AMD, retinitis pigmentosa) or retinal vascular disease (diabetes, vein occlusion); primary outcome measures: adverse events	Park *et al.*, 2012 [112]; NCT01736059
Multi center USA; Stem Cells, Inc., Newark, California, CA, USA	2012/completion 2015 (estimated)	human central nervous system stem cells (HuCNS-SC)	▪ unilateral transplantation into sub-retinal space through standard surgical approach in patients with advanced atrophic AMD; primary outcome measures: adverse events	McGill *et al.*, 2012 [113]; NCT01632527
Rubens Siqueira Research Centre, São Paulo, Brazil; University of Sao Paulo, São Paulo, Brazil	2011/completion January, 2014 (estimated)	autologous BMSC	▪ intravitreal injection in patients with advanced AMD (atrophic or neovascular); primary outcome measures: change in visual acuity	Siqueira *et al.*, 2011 [114]; NCT01518127

## 7. Conclusions

Retinal degenerative diseases, in particular highly prevalent diseases, such as AMD, with a high risk of losing vision, claim a tremendous societal burden in terms of quality of life, decrease in productivity and healthcare expenditures [79]. Consequently, there is an urgent medical need to advance strategies for understanding their pathophysiologies and for establishing valid platforms for rapid therapeutic developments. Stem cell-based disease modelling is a novel and rapidly advancing field with apparently unlimited potential to meet those demands. A number of proof-of-concept studies have been published and have further underscored the advancements and the power of stem cell technology [47,79,115], heralding a new era in biomedical research, as well as drug discovery and development.

Of particular interest are the opportunities in the field of personalized medicine. Patient-derived hiPSCs and their tissue-specific derivatives may be used to individually identify and test drugs for their effectiveness in a complex genetic environment. Furthermore, stem cell-based replacement therapies could be tailored to the patients’ needs, although the immunological advantageous of autologous cell transplantation may be lost unless the harmful genetic constellation of the donor’s cells can be corrected. GMP-grade cells for transplantation are available, and the first clinical applications for RPE cell replacement are under way [110]. Based on these visionary developments, which build upon major technical innovations in stem cell research, we trust to see light at the end of the tunnel in the near future.
